# The Effect of the COVID-19 Pandemic on Odontogenic Cervicofacial Infections in a Single Center in Greece

**DOI:** 10.7759/cureus.61333

**Published:** 2024-05-29

**Authors:** Alexandros Louizakis, Dimitris Tatsis, Konstantinos Paraskevopoulos, Asterios Antoniou, Athanasios Kyrgidis, Konstantinos Vahtsevanos

**Affiliations:** 1 Department of Oral and Maxillofacial Surgery, George Papanikolaou General Hospital, Aristotle University of Thessaloniki, Thessaloniki, GRC

**Keywords:** covid-19 pandemic, dental abscess, odontogenic infections, intensive care unit admission, cervicofacial infections

## Abstract

Introduction: Odontogenic cervicofacial infections are still an ongoing problem, requiring immediate hospital admittance and management. The aim of this study is to reflect the number of patients with cervicofacial infections who were admitted during the coronavirus disease 2019 (COVID-19) pandemic period in a single, point of reference center in Northern Greece as well as analyze the quantitative and qualitative parameters of patient characteristics and management data.

Methods: This was a retrospective cohort study that included all the patients with cervicofacial infections who were admitted to our unit during the COVID-19 pandemic, specifically between 2020 and 2021. For comparative reasons, patients admitted with cervicofacial infections between 2019 and 2020 (pre-COVID period) were analyzed.

Results: In total, 341 patients fulfilled the criteria for this study. Specifically, the number of admitted patients was 151 in the pre-COVID era instead of 190 patients in the pandemic. The mean age of the patients was 45.3 years, with a slight male predominance (54.7% males to 45.3%). The mean duration of hospitalization was 2.5 days in the pre-COVID period instead of 3.42 days in the pandemic. Interestingly, in the pandemic, eight times more patients were admitted to the ICU post-operatively, in contrast to the pre-COVID period (23 vs 3 patients). Also in the COVID period, almost 54.9% of the patients presented with fever and 49.6% with trismus. Moreover, the submandibular space involvement was the most common space of infection in both COVID and pre-COVID groups with (58.9% and 49.7%) respectively. In one-third of all cases, a post-extraction infection of a third molar was the main cause of abscess.

Conclusion: Cervicofacial infections during the COVID-19 pandemic appeared with more severe symptoms and resulted in an increased number of patients who needed admittance to the intensive care unit, in contrast to the pre-COVID era. Also, the mean length of stay was increased for a day at the same period. This study could be used as an example for further research, in case of similar pandemic situations in the future.

## Introduction

The most common cause of cervicofacial infections is odontogenic [1-3]. Despite the new treatment methods, the evolution of antimicrobial drugs, and the major preventive campaigns in oral and dental health, odontogenic infections are still considered a common phenomenon, even in developed countries. These infections evolve fast, easily spread to deeper cervical spaces, and can have serious, possibly life-threatening, complications [1,4-7].

Odontogenic cervicofacial infections can appear with varying symptomatology depending on their severity, such as trismus, dyspnea, dysphagia, cardiopulmonary complications, airway and tongue displacement, mediastinitis, compromised airway, mediastinitis, Lemierre’s syndrome, sepsis, tissue necrosis, and more [6,7].

The teeth, mostly associated with infections and their spread to deeper cervicofacial spaces, are the mandibular molars and most frequently the third molars [2,3,8,9]. Delayed definitive care by health professionals who may underestimate an arising infection, or patients’ negligence to present themselves to emergency facilities when an infection manifests, may lead to life-threatening situations, even death [1,6,10,11].

In Greece, the coronavirus disease 2019 (COVID-19) pandemic, which appeared in March 2020, had a significant impact on the number of patients who were admitted to hospitals with severe cervicofacial infections. The difficulties in seeking and acquiring professional health care during the pandemic, the government’s instructions for the public to stay home, the “urgent care services” such as remote telephone triage or advice often applied to patients, combined with the fear of transmitting the COVID-19, constitute the main reasons why patients arrived late at the emergency unit, having developed symptoms of advanced odontogenic infections [6,8,10,12,13].

The purpose of this study is to evaluate the scale of the COVID-19 effects on the quantitative characteristics and the qualitative data of admissions to a single center-point of reference hospital in Northern Greece during that period, in comparison to the pre-COVID era, while assessing the number of admissions to the ICU, as well as the total length of hospitalization and ICU stay.

## Materials and methods

This study is a retrospective analysis of patients hospitalized in one single center (George Papanikolaou General Hospital, Thessaloniki). Ιt was performed in accordance with the Declaration of Helsinki and approval from the local scientific committee was not required due to the retrospective evaluation of anonymized medical data.

The study included all patients admitted to the Department between March 2019 and February 2021 with an acute odontogenic cervicofacial infection. Patients were divided into two distinct groups: the first consisting of patients during the period March 2019 to February 2020 (pre-COVID period) and the second consisting of patients during the period March 2020 to February 2021 (COVID period). Patients with the same diagnosis admitted to the ICU were also considered in the study. No patients diagnosed with an acute odontogenic cervicofacial infection were excluded from the study during the above-mentioned periods.

The recorded parameters were as follows: demographics, clinical presentation, definitive treatment delays, medical comorbidities, length of stay, need for intensive care, and outcomes. The need for antimicrobials and surgical drainage was also recorded. A comparison of these parameters occurred for both groups.

All separate variables have been incorporated in the analysis. Categorical data were presented as numbers and frequencies and were compared using Pearson’s chi-square test. The Type I error probability associated with all tests in this study was set to 0.05. Statistical analyses were performed using IBM SPSS Statistics for Windows, Version 24 (Released 2016; IBM Corp., Armonk, New York, United States).

## Results

In total, 341 patients were selected and included in both the COVID and the pre-COVID era. All variables of the study are summarized in Tables 1-3. Of the 341 patients, 151 were admitted during the pre-COVID period, whereas 190 were admitted during the COVID period (namely 39 more patients), p<0,001. The average age was 44.6 years during the pre-COVID period and 45.6 years during the pandemic. A slight male predominance was observed during the pre-COVID era (54.7% male vs 45.3%), whereas during the COVID era, a female predominance was noticed (45.6% female vs 54.3% male). Eleven percent of all patients had a type of major medical comorbidity (9.9% vs 11.6% complication rates in the two groups respectively) (Table 1).

**Table 1:** Patients’ demographics and medical comorbidities

Period	Pre-COVID	COVID	Total
Number of patients	151	190	341
Mean age (years)	44.6	45.6	45.3
Gender distribution (Male/female, %)	54.7/45.3	45.6/54.3	48.3/51.7
Smokers (%)	45	33.1	38.3
Diabetes (%)	3.3	3.3	3.2
Other major comorbidities (%)	9.9	11.6	10.8

**Table 2:** Patients’ clinical characteristics at the time of admission

Period	Pre-COVID	COVID	Total
Clinical characteristics (%)			
Fever (>38^o^C)	66.2	49	56.6
Trismus	33.1	49.6	42.3
Primary manifestation (%)			
Submandibular area	58.9	49.7	53.9
Ludwig’s angina	1.3	5	3.4
Other	39.8	45.3	42.8
Aetiology (%)			
Post-extraction	32.5	33.1	32.8
Third molar involvement	31.1	26.5	28.4
Other	36.4	40.4	38.6

**Table 3:** Length of stay

Period	Pre-COVID	COVID	Total
Mean total length of stay (days)	2.5	3.42	3.1
Number of patients admitted to the ICU	3	23	26
Mean length of stay in the ICU (days)	2.5	3.42	3.1
Complications	0	0	0

More than half of the patients developed fever (54.9% in the total cohort), while trismus was evident in 33.1% of the patients during the pre-COVID period and 49.6% of the patients during the COVID period. Involvement of the submandibular space was the most common infection in both pre-COVID and COVID groups (58.9% and 49.7% respectively). Infections related to post-extraction procedures or involving the third molar were the most common causes of admission (Table 2).

The mean length of stay was 2.5 days during the pre-COVID period vs 3.42 days during the COVID period, namely one additional day of hospitalization was evident during the COVID era, as well as an average length of stay of 3.1 days in total, both in the oral and maxillofacial surgery (OMFS) department and the ICU (2.5 vs 3.42 days respectively), p=0.07 (Table 3).

Particularly important was the fact that the patients who post-surgically had to be admitted to the ICU were almost eight times more during the COVID period, contrary to the pre-COVID period (23 vs 3 patients respectively), p<0,001. Similar was the case for patients who were hospitalized with severe deep neck infections during the COVID period (23 vs 3 patients), also showing an eight times higher frequency compared to the pre-COVID period.

## Discussion

This retrospective study was designed to highlight the fact that between the years 2019 and 2020, the COVID-19 pandemic greatly affected the patients who were admitted to the hospital with severe cervicofacial infections [10]. The patients’ anxiety about not transmitting COVID-19, as well as the governmental instructions to stay home and avoid unnecessary visits to the hospital to secure the national health care system, led to a prolonged stay of the patients at home with persisting symptoms of cervicofacial infection. As a result, they were deterred from attending the hospital at the early stages of infection, thus deteriorating their clinical manifestation when presented at the emergency unit [6,10,14,15].

The mandibular molars and especially the third molars of the lower jaw were the primary cause of advancing infection at a leading percentage [3,5,8,10,12,14,16,17].

Poor oral health in Greece was extrapolated by the restriction of access to primary dental care due to the pandemic, leading to an increase in patients requiring hospitalization and high-dependency care [5,6,15]. As indicated in this study, eight times more patients presented at the OMFS clinic with signs and symptoms of severe deep neck infections (23 patients during the COVID period versus 3 during the pre-COVID period).

Once an odontogenic infection spreads to deeper cervicofacial spaces, a series of complications may easily occur, the most serious of them being airway displacement and obstruction, often needing tracheostomy and descending necrotizing mediastinitis. This may cause a serious complication if the pus finds its way through the thoracic cavity via the prevertebral or “danger space”, the large neck vessels (internal jugular vein-IJV, carotid artery), or the subdermal. This constitutes an urgent situation requiring immediate thoracotomy and drainage [8]. Other major complications are cardiopulmonary ones, as well as Lemierre’s syndrome describing the thrombophlebitis of the IJV which can also cause metastatic emboli to other organs and the lungs [2,3,7-10,15,18-20].

As regards the number of patients who were admitted with cervicofacial infections, a major increase was identified in this study. The patients were twice as many as those in the pre-COVID period (302 vs 151). This is explained by the fact that the majority of cervicofacial infections were odontogenic ones. In most cases, the mandibular molars and especially the third molars of the lower jaw were the primary cause of an advancing infection.

Consequently, even more patients were admitted to the emergency unit with the above-mentioned symptoms, which would otherwise be prevented at a primary stage with just a check-up at the dentist [6,8,17]. A study by Kün-Darbois et al. reported a 44% decrease in the number of patients who were admitted with odontogenic neck cellulitis during the COVID period, a finding, however, that could not be thoroughly explained by the same authors [21].

Furthermore, the average age of the patients was between 44 and 45 years during both periods. A slight male predominance was reported during the pre-COVID period with a percentage of 54.7%, in contrast to the COVID period during which females held the first place with a percentage of 54.3%. This could only be explained by the fact that women tend to be more self-conscious about their health and consult specialists more compared to males. The majority of the other studies in both periods present a clear male predominance in all cases of patients with cervicofacial infections [2,5,9,10,12,16].

Another clinical finding relates to the length of stay in the OMFS clinic which was raised by a day during the COVID period compared to the pre-COVID one (3.42 days vs 2.5 days respectively). The reason for this is that during the COVID period, patients persisted more with their symptoms and arrived later in the emergency unit when the odontogenic infection had already been spread and multi-space involvement had already been installed. Thus, patients arrived with much more severe symptomatology such as trismus, dysphagia, and breathing problems requiring, apart from immediate surgical intervention, higher doses of antimicrobial therapy and, in specific cases, admission and prolonged stay at the ICU. In most cases, a surgical reintervention was mandatory. Therefore, the total hospital stay was increased [2,6,8,9,14,15].

On the other hand, according to Johnson et al., no significant differences were reported concerning the total length of stay during both periods, while in the article of Politi et al., a reduction was found since, as mentioned, most of the patients received a more decisive treatment with faster intervention and surgery, as well as with an increase in extraoral drainage. In any case, the immediate treatment and surgical intervention, when needed, had a significant influence on the reduction of the total hospital stay [10,12,16].

Particularly interesting was the fact that a great number of the patients who were hospitalized for cervicofacial infection during the COVID period and underwent surgical intervention need to be admitted to the ICU. More specifically, the number was eight times higher during the COVID period (23 patients vs 3 patients during the non-COVID era). Moreover, the average length of stay in the ICU was increased by a day during the COVID period (3.42 vs 2.5 days). As already mentioned, a cervicofacial infection with multi-space involvement is a major factor for ICU admittance.

Deep neck infections often correlate with major complications such as trismus, airway displacement, neck dyskinesia, and mediastinitis, which require admittance and often prolonged stay in the ICU [5,9,11,22]. In addition, in cases of patients with major comorbidities such as diabetes, cardiovascular diseases, lung disease, endocrinopathy, malignancies, and immunosuppression, the length of stay in the ICU is usually increased. Moreover, patients who do not show clinical improvement require reintervention, which may prolong their stay in the ICU [4,6,23].

Another factor that may increase the patients’ stay in the ICU is the prolonged intubation instead of tracheotomy, when required. According to the general literature, tracheotomy, when required to secure the airway, may dramatically reduce the total length of stay in the ICU and hospitalization in general [8,24].

This study has several limitations. At first, this is a retrospective study with all limitations owing to its design. Moreover, the number of patients included is limited, while the generalization of the results would be statistically wrong. However, these patients were admitted to the hospital at a very advanced stage of the infection and often with severe symptoms, mostly due to their difficulty in seeking, and therefore acquiring, primary health care during the pandemic.

## Conclusions

The present study aims to reveal the major issue of odontogenic infections that still concern a substantial number of patients worldwide. Specifically, during the COVID-19 pandemic, the prolonged stay of patients at home with symptoms of cervicofacial infections because of the exceptional governmental measures impeding them from seeking primary health care led to more serious cervicofacial infections. By the time they were admitted to the hospital, the abscesses had already been spread to deeper cervicofacial areas, the infections had already become more serious and patients needed not only to be hospitalized for longer periods but also to be admitted to the ICU for extended periods. In the present study, almost eight times more patients were admitted to the ICU during the pandemic, the average length of stay in the hospital was increased by at least one day and the total number of patients who were admitted to the hospital was much higher than that during the pre-COVID period.

This study could be used as an example and as a basis both for further research and for faster response in possible future pandemics, to reduce the impact of cervicofacial infections occurring mostly due to odontogenic reasons. The general population should be educated and better informed about the prevention of odontogenic infections and more frequent dentist appointments should be strongly encouraged. Moreover, General Practitioners should be more aware of the OMFS specialty and its clinical spectrum, to refer patients correctly and faster in cases of cervicofacial infections, mostly occurring due to odontogenic reasons. In conclusion, the public health system should derive lessons from these challenging times, utilizing the COVID situation as a precedent to embrace new methods of preventing similar cases, thereby reinforcing its resilience and adaptability for the future.
